# Implementation of evidence-based parenting programs under real-world conditions: Protocol for a scoping review

**DOI:** 10.1371/journal.pone.0256392

**Published:** 2021-08-19

**Authors:** Rita Pinto, Catarina Canário, Orlanda Cruz, Maria José Rodrigo

**Affiliations:** 1 Faculty of Psychology and Education Science, University of Porto, Porto, Portugal; 2 Faculty of Psychology, University of La Laguna, San Cristóbal de La Laguna, Spain; Public Library of Science, UNITED STATES

## Abstract

Protecting children is recognized as a public health priority and supporting parents through the implementation of evidence-based programs is a well-known strategy to achieve this. However, researchers highlight that these programs remain insufficiently implemented in real-world contexts. A knowledge gap exists between the intended implementation of evidence-based parenting programs and their actual implementation on real-world dynamics. This scoping review aims to provide a comprehensive understanding of how evidence-based parenting programs have been implemented under real-world conditions by providing a map of available evidence and identifying knowledge gaps. The overall research question is: "How have evidence-based parenting programs been implemented under real-world conditions?". The proposed scoping review follows the framework originally described by Arksey and O’Malley, Levac and colleagues, and the Joanna Briggs Institute: (1) identifying the research questions; (2) identifying the relevant studies; (3) study selection; (4) charting the data; (5) collating, summarizing, and reporting the results; (6) consultation. The Preferred Reporting Items for Systematic reviews and Meta-Analyses extension for Scoping Reviews (PRISMA-ScR) will inform the search strategy. The results will be described in relation to the research questions and in the context of the purpose of the review. This scoping review will help to bridge the implementation gap between research evidence and its translation into practice.

## Introduction

Respect for children’s rights is one of the top priorities of public health and is ensured by a strong commitment to child protection [1]. Research has proven that the family is the primary context for children’s well-being and healthy development, and growing awareness has been devoted to the need to support parents in fulfilling their childcare responsibilities [2]. Evidence-based parenting programs (EBPP) effectiveness in addressing child behavioral problems and improving parenting practices has been established [2, 3]. As a result, practices based on the best research evidence are increasingly encouraged by the scientific community [4]. There has been increasing recognition of the importance of incorporating these empirically supported practices into the daily services attending children and families [5]. This necessity is motivated by the belief that prevention efforts should be guided by science and that interventions shown to be effective through rigorous evaluation should be used [6].

Prevention scientists distinguish between efficacy and effectiveness studies [7]. Efficacy represents the positive effects of a program under optimal implementation conditions, whereas effectiveness refers to the effects of a program implemented under real-world conditions [7]. However, the results of EBPP efficacy studies often are not replicated when these programs are transferred to widespread implementation in communities [8]. Despite its high relevance, the question of efficacy provides little insight into how best to implement the intervention in different real-world settings [9]. Indeed, efforts to implement interventions under real-world conditions are typically much less effective than outcomes achieved through randomized clinical trials in laboratories [9]. The positive outcomes achieved in controlled research settings are not always replicated in natural settings within mainstream services [7, 10]. While it is of great relevance to use EBPP in real-world settings [11], the transition of these programs from the initial trial stage to real-world implementation is both complex and poorly understood [6]. There remains a gap in translating the success of programs in efficacy trials into natural settings [11]. The implementation process influences the outcomes achieved in parenting programs [9]. Therefore, the evaluation of implementation can contribute to researchers’ knowledge of whether an intervention failed because it was ineffective or implemented incorrectly [12].

Implementing EBPP in the real world is an essential step in translational research to make effective programs widely available to improve public health [13]. Some EBPP have attracted international investment beyond their original place of development and have been widely disseminated and implemented in various countries. Examples include EBPP of the Triple P System [14] and the Incredible Years Program [15]. Nevertheless, some researchers highlighted that evidence-based approaches remain insufficiently available in many key services for children and families delivered in the community [16, 17]. Today, practitioners can access many registry tools that evaluate programs using evidence criteria [18]. However, our knowledge of published studies examining EBPP inclusion in real-world settings [19] and barriers and facilitators to EBPP implementation in these settings is limited [20]. In 2016, a special issue with a collection of articles was dedicated to the identification, description, and evaluation of some EBPP implemented in real settings in Spain [12]. The need to expand this work is acknowledged, highlighting the relevance of developing further reviews of EBPP implemented under real-world conditions [12].

Although there is a global interest in the field of Implementation Science [21, 22], high-quality implementation of programs in real-world contexts is acknowledged as a significant challenge [5]. The many complexities involved hinder the study of the implementation process in detail [23]. Notwithstanding the complexity of designing and conducting implementation studies, reviewing the implementation process is an essential feature of program evaluations [12, 21]. In order to increase knowledge about the best policies and practices related to specific interventions, settings, populations, and conditions, it is essential to evaluate the implementation process [12]. The studies that focus on implementation provide information about the feasibility of a particular EBPP and the effort required to implement it effectively under real-world conditions [12]. For all these reasons, the implementation process is now recognized as a quality standard for EBPP [21, 22, 24]. There is a growing awareness that implementation research is critical to the adoption, replication, and scale-up of EBPP [25].

In general, prevention science research has moved from identifying the efficacy of programs to evaluating their effectiveness under real-world conditions [7]. However, while research on effective practices continues to increase, research on implementation is lacking [9]. Consequently, a knowledge gap remains between how EBPP should be implemented and how they are actually implemented in the real world [26, 27]. Therefore, researchers now recognize that the focus should not only be on measuring program outcomes ("Did it work?"), but that attention needs to be paid to the evaluation of the implementation process ("Under what conditions?") [24]. However, researchers highlight that there is not yet agreement on what specific implementation information should be examined and reported in studies [24]. Some suggestions for key implementation components include fidelity (i.e., adherence to the program model, content, and dosage), quality of delivery (i.e., the skill with which practitioners conduct program sessions and interact with participants), and program adaptation (i.e., changes made to the program) [28].

Much has been written about the lack of knowledge in the transition from research to practice [19]. Nonetheless, as far as can be determined, this would be the first time a review has been written with an implementation lens to map the evidence in the field of EBPP implemented under real-world conditions. Scoping reviews can be particularly useful for collecting literature in areas of emerging evidence [29], such as Implementation Science. To this end, a scoping review will be conducted to establish a comprehensive understanding of how EBPP have been implemented under real-world conditions, providing a map of the available evidence and identifying knowledge gaps.

## Materials and methods

The proposed scoping review is guided by the six-step framework originally described by Arksey and O’Malley [30], which was later reviewed, endorsed, and extended in the methodological literature by Levac and colleagues [31] and the Joanna Briggs Institute [29]. The stages of the review include: (1) identifying the research questions; (2) identifying the relevant studies; (3) study selection; (4) charting the data; (5) collating, summarizing, and reporting the results; and (6) consultation. Each phase is described in more detail below in line with the objectives of the current scoping review. The last literature search will take place on 31 December 2021.

This protocol has been registered at the Open Science Framework (available at: osf.io/4fydu).

### Stage 1: Identifying the research questions

The proposed scoping review aims to establish a comprehensive understanding of how EBPP have been implemented in real-world settings, providing a map of the available evidence and identifying knowledge gaps. Based on the PCC mnemonic (population, concept, and context), the main research question is as follows: How have evidence-based parenting programs been implemented under real-world conditions?

The research sub-questions are:

- What evidence-based parenting programs have been implemented in real-world settings?
- What are the target populations (parents and children) receiving these programs?
- In what settings are these programs offered?
- What information is reported about implementation in studies of EBPP that have been conducted under real-world conditions?
- What are the facilitators and barriers to the implementation of EBPP encountered in these contexts?

### Stage 2: Identifying relevant studies

The second stage of the scoping review process will establish the criteria for selecting studies for inclusion and exclusion in the review. Although a scoping review is designed to cover a wide range of literature, these criteria will guide the search and help to filter out relevant sources. The search strategy will be as comprehensive as possible to identify both published and unpublished (grey) literature.

The review process will include three levels of screening: (1) title review, (2) abstract review, and (3) full-text review. As recommended in JBI reviews [29], a three-step search strategy will be used. The first step will be an initial limited search of two appropriate online databases relevant to the topic, Academic Search Ultimate and Scopus. Initially, the following search terms will be used: implement*, evidence-based program, parent*, community, or real-world. The wildcard character (*) will be used to ensure that variations of each keyword are found. Following this initial search, the text words in the title and abstract of the retrieved papers, as well as the index terms used to describe the articles, will be analyzed.

A second search with all identified keywords and index terms will then be performed in all included databases: APA PsycArticles, APA PsycBooks, APA PsycInfo, BMC Medicine, Education Source, ERIC, Fonte Académica, MEDLINE, PLOS ONE, Psychology and Behavioral Sciences Collection, PubMed, Sociology Source Ultimate, SAGE Research, and Web of Science. Duplicates will be excluded. The references list of sources selected from the full-text will then be screened to look for additional sources and ensure that relevant articles are included in the scoping review. Potentially relevant unpublished (grey) literature will be identified through targeted searches of dissertations/ theses (ProQuest Dissertations & Theses Global), relevant websites, study registries and reports, and conference abstracts. Only English, Spanish, French, and Portuguese publications will be included as resources for translating studies into other languages are not available. Date limits will not be applied.

The search strategy will be piloted independently by at least two authors to verify the appropriateness of the keywords and databases. An experienced researcher on the topic will be consulted for the contribution of specific search terms. Considering that database search is one of the core elements of the search plan, and it should be peer-reviewed, we will use the Peer Review of Electronic Search Strategies (PRESS) checklist [32] to guide and improve the quality and comprehensiveness of the electronic literature search strategy.

### Stage 3: Study selection

**Fig 1:**
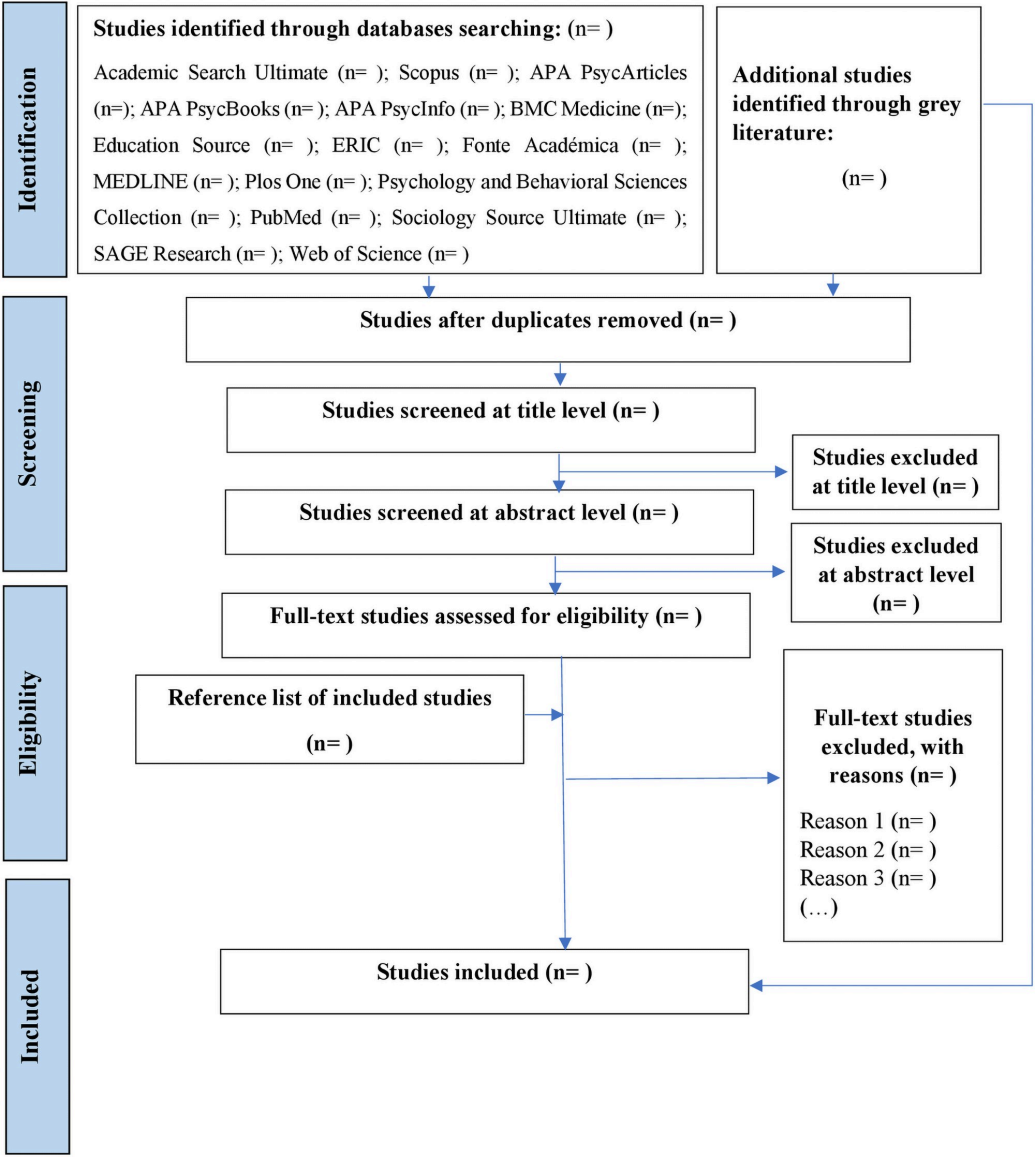
PRISMA flow chart for the scoping review process. Adapted from: Moher, Liberati, Tetzlaff, and Altman [36].

PCC mnemonics will guide the screening of each title and abstract. Other eligibility criteria will ensure that the content of included studies is relevant to the research question. Search terms will be determined in consultation with the research team. The searches will continue until saturation is reached and no new studies meeting the inclusion and exclusion criteria are identified. The primary author and one of the co-authors will conduct independent assessments of the full-text articles to determine whether they meet the inclusion/exclusion criteria. Any disagreements will be discussed between the two reviewers until consensus is reached, or by arbitration of a third reviewer. Reasons for exclusion will be noted, and the process of study selection will be documented in a flow chart (Fig 1), according to Preferred Reporting Items for Systematic reviews and Meta-Analyses extension for Scoping Reviews (PRISMA-ScR) [33]. The electronic database search will be recorded in a table, as well as the characteristics of the included studies. The software used to manage references and search results will be Zotero [34] and Rayyan [35], respectively.

### Inclusion criteria

- Type of participants: this review will include all research studies of EBPP targeting parents of children aged 2–16 years. The age range was set between 2 and 16 years old to have studies including preschool, school-aged children, and young adolescents.
- Concept: the core concept examined by the scoping review is the implementation of EBPP.
- Context: EBPP should be implemented in real-world settings.

### Exclusion criteria

- Given that the proposed review explores evidence-based programs for parents, studies that primarily target foster/adoptive parents or children in extended family placements will be excluded.
- Clinical/explanatory trials or studies not applied under real-world conditions will be excluded. This review must map real-world evidence and include data collected in different natural contexts.

### Stage 4: Data charting

The research team will develop a data extraction form to confirm the study’s relevance and extract the study features. Study characteristics to be extracted will include: author(s), title, year of publication, type of source, name of the program used, country of origin and country where the program was implemented, population characteristics, the context in which the program was delivered and by whom, implementation characteristics described for each program, reported facilitators and barriers. Attempts will be made to contact the authors of the included papers if key information is missing or unclear.

To ensure a systematic data extraction process, the research team will design an extraction spreadsheet using Microsoft Excel. Before beginning the extraction process, the spreadsheet will be tested on 10 randomly selected studies to ensure that it correctly captures the relevant information. Then the form will be refined accordingly. Charting the results can be an iterative process where the spreadsheet will be continually updated [30]. To ensure accurate data collection, the extraction of relevant article characteristics will be performed independently by two reviewers. Any discrepancies will be discussed further to ensure consistency.

### Stage 5: Collating, summarizing, and reporting the results

It is important to note that scoping reviews do not synthesize the results of the included evidence sources, as this should instead be done as part of conducting a systematic review. Therefore, the results of the included sources will be described in relation to the research questions and in the context of the overall aim of the review. Also, the aggregated findings will provide an overview of the research rather than an assessment of the quality of individual studies.

Due to the potential scope and heterogeneity of the material included in the scoping review, it is not possible to determine in advance the optimal method for collating and reporting the results. This will be refined during the review process as reviewers become more familiar with the content of all included sources [29]. Therefore, a narrative report and/or a visual form (e.g., tables and charts) will be produced, as appropriate, to summarize the extracted data.

### Stage 6: Consultation

An experienced researcher will advise throughout the process, including in the planning, execution, and dissemination of the review.

## Discussion

The ultimate goal of the proposed scoping review is to identify, characterize, and summarize the evidence on the topic under review, including the identification of knowledge gaps. This study protocol describes a detailed plan for conducting the review, including background, objectives, and methodology. Publishing this protocol will ensure the reproducibility of the study and benefit from expert review feedback.

A comprehensive literature search will be conducted across multiple electronic databases, using a methodological framework to chart and map findings, and present a discussion of knowledge gaps and future research opportunities. The purpose of the scoping review is to provide an overview of the existing evidence, and as such, the methodological assessment to determine the quality of the data retrieved from the selected studies will not be performed.

Over the past few decades, the field of implementation research has grown considerably, resulting in increased interest from researchers, funders, practitioners, and policymakers [37]. Despite its potential to advance science, limited research has focused on mapping issues in this field [38]. Therefore, by mapping evidence in the field of real-world implementations of EBPP, this scoping review will help to bridge the implementation gap between research evidence and its translation into practice.

Any changes to the study protocol will be reported in the scoping review, as well as the discussion of the limitations of the scoping review process.

The findings of the proposed review will be disseminated through relevant stakeholder groups (e.g., professionals providing family and parenting support) and presentations at relevant scientific meetings. In addition, an article outlining the results of the scoping review will be submitted for publication in a scientific journal.
