# Correction: Implementation of evidence-based parenting programs under real-world conditions: Protocol for a scoping review

**DOI:** 10.1371/journal.pone.0292826

**Published:** 2023-10-09

**Authors:** Rita Pinto, Catarina Canário, Orlanda Cruz, Maria José Rodrigo

There are errors in the Funding section. The correct Funding statement is: This work was supported by a Ph.D. studentship (2020.04778.BD) funded by the Portuguese Foundation for Science and Technology (FCT) and by the European Social Fund (ESF) attributed to Rita Pinto, and also by national funding from the Portuguese Foundation for Science and Technology (UIDB/00050/2020). FCT’s website: https://www.fct.pt/index.phtml.en; ESF’s website: https://ec.europa.eu/esf/home.jsp?langId=en. The funders had no role in study design, the decision to publish, or preparation of the manuscript.
